# Therapeutic pulpotomy for permanent teeth with irreversible pulpitis: comparative results from a practice-based quick poll in the USA and UK

**DOI:** 10.1038/s41405-026-00404-5

**Published:** 2026-02-02

**Authors:** Thibault NE Colloc, David NJ Ricketts, Janet E. Clarkson, Ashraf F. Fouad, Craig R. Ramsay, Joana Cunha-Cruz

**Affiliations:** 1 University of Dundee, Scotland, Dundee, UK; 2 University of Alabama at Birmingam, AL, USA; 3 University of Aberdeen, Scotland, Aberdeen, UK

**Keywords:** Dental epidemiology, Pulp conservation

## Abstract

**Background/Aims:**

Treatment paradigms for teeth with signs and symptoms of irreversible pulpitis in permanent teeth are evolving, with increasing interest in conservative approaches such as therapeutic pulpotomy. Understanding the perspectives of both general dentists and endodontists is essential to defining current clinical practices and informing future research. This study explored dental practitioners’ approaches to the diagnosis and management of irreversible pulpitis, with a focus on the use of pulpotomy as a definitive treatment.

**Methods:**

An online “Quick Poll” cross-sectional survey was distributed to dental practitioners in the United States via the National Dental Practice-Based Research Network and in the United Kingdom through open online channels, including local dental networks, practice-based research networks, and social media platforms.

**Results:**

A total of 750 practitioners responded (USA: 416; UK: 334), most of whom were general dental practitioners with over 10 years of experience. Irreversible pulpitis was most diagnosed in 1–5 patients per month. Root canal therapy was the predominant treatment in both countries (USA: 77%; UK: 90%), with extraction frequently selected in the UK (50%). Pulpotomy was reported as a treatment strategy by 20% of USA and 16% of UK respondents, though a larger proportion expressed willingness to consider it as a definitive option (USA: 47%; UK: 87%).

**Conclusion:**

This preliminary study highlights the growing interest in therapeutic pulpotomy as a definitive treatment for teeth with signs and symptoms of irreversible pulpitis in permanent teeth in primary care. Differences in clinical adoption and attitudes between the USA and UK suggest opportunities for further research, education, and implementation support to facilitate the integration of vital pulp therapies into routine practice.

## Introduction

Untreated dental caries can often cause pain, tooth loss, spreading endodontic infections, and even mortality [1, 2]. When a carious lesion extends near the dental pulp, symptoms may arise, which are often diagnosed as symptomatic or asymptomatic irreversible pulpitis [3]. Historically, the treatment options for irreversible pulpitis were limited to an extraction or a root canal treatment (RCT). However, when the damage and inflammation of the pulp are limited to its coronal part, it is advantageous to preserve the radicular part of the pulp vitality with a minimally invasive procedure [3].

A therapeutic pulpotomy is a vital pulp therapy (VPT) procedure involving the removal of inflamed coronal pulp tissue and placement of a biocompatible material—typically a calcium silicate-based cement (CSC)—directly onto the remaining vital pulp. It may be performed as a partial or full pulpotomy, depending on the extent of inflammation and ability to achieve haemostasis [4]. Unlike emergency pulpotomy, often used in conjunction with the placement of calcium hydroxide or a steroid and antibiotic-based paste (i.e. ledermix; odontopaste), which provides temporary symptom relief until definitive treatment can be provided (root canal treatment or extraction) [5], therapeutic pulpotomy using CSC is always intended as a definitive procedure.

Accurate pulpal and periapical diagnosis is essential for the success of therapeutic pulpotomy. Symptomatic irreversible pulpitis is typically diagnosed based on clinical symptoms such as spontaneous or prolonged pain following stimulation. Normal apical tissues are confirmed by the absence of pain on percussion and palpation, and no radiographic signs of apical pathosis, as data show that preoperative radiographic signs of apical periodontitis are a significant predictive factor of failure of vital pulp treatments in the long term [6]. Final confirmation of diagnosis is made upon pulp exposure, where the presence of bleeding tissue in all canals, without purulence and with haemostasis achieved within 10 min, supports proceeding with pulpotomy. If these criteria are not met, pulpectomy and root canal treatment may be indicated [7].

This technique is recommended, in restorable permanent teeth with signs and symptoms of irreversible pulpitis, by both the American Association of Endodontists (AAE) and the European Society of Endodontology (ESE) [4, 8, 9].

One systematic review of cases with spontaneous pulpal pain showed comparable patient-reported pain outcomes between pulpotomy and RCT in the short term, with some trials reporting equivalent clinical success over extended follow-up periods [10]. More recently, the comprehensive systematic review and meta-analysis by Coll et al. has reinforced the effectiveness of vital pulp therapy across a range of diagnostic categories, including symptomatic irreversible pulpitis [11]. Their findings indicate high success rates for indirect pulp treatment, direct pulp capping, partial pulpotomy, and full pulpotomy (91% to 97% success with 12 to 36 months follow-up), with no significant differences in outcomes when calcium silicate-based materials are used. A multicentred randomised controlled clinical trial in the UK funded by the National Institute for Health and Care Research (NIHR) is currently underway [12].

The National Dental Practice-Based Research Network (the Network) in the USA was founded in 2012 with the mission to improve oral health by conducting dental practice-based research and by serving dental professionals and their patients through education and collegiality [13, 14]. Dental professionals who practice in the USA can become members of the Network and engage with academics in the development and implementation of studies that are of direct interest to them and their patients, and by incorporating findings from these studies into their daily clinical practice [15].

Similar practice-based research networks exist in the United Kingdom across its constituent nations, such as the Scottish Dental Practice-Based Research Network and the Northern Dental Practice-Based Research Network in England. These networks foster collaboration among primary care dental practitioners and support the development of evidence-based practice through large-scale clinical research. Notable examples include the SCRIPT (Selective Caries Removal in Permanent Teeth) and PIP (Pulpotomy in Permanent Teeth with Irreversible Pulpitis) studies [12, 16]. This ecosystem is further supported by national guideline development bodies such as the Scottish Dental Clinical Effectiveness Programme (SDCEP) and the National Institute for Health and Care Excellence (NICE), which translate research findings into clinical recommendations. Like the National Dental Practice-Based Research Network in the USA, UK networks enable clinicians to engage in research that is directly relevant to their practice, promoting the integration of new evidence into routine care and improving patient outcomes.

To explore practice patterns in the clinical management of irreversible pulpitis in permanent teeth, a brief 5-question scenario-based “Quick Poll” was distributed to Network members in the USA. In parallel, a similar poll was disseminated in the United Kingdom via an open online survey, shared through local dental networks, practice-based research networks, and social media platforms. This international approach aimed to capture perspectives from general dentists and endodontists in both countries regarding the use of therapeutic pulpotomy with calcium silicate-based materials as a conservative alternative to root canal treatment. The findings will inform the design of a future clinical study evaluating the effectiveness of pulpotomy in primary care settings.

## Materials and methods

### Study design and participants

This study consisted of a cross-sectional survey exploring the clinical management of irreversible pulpitis in permanent teeth. In the United States, a 5-question “Quick Poll” was distributed to dental practitioners (*n* = 4483) who were members of the National Dental Practice-Based Research Network. The poll aimed to gather preliminary data on current beliefs and behaviours in primary care, particularly regarding the use of pulpotomy as a conservative alternative to root canal treatment. Quick Polls are a low-cost, rapid engagement tool used by the Network to inform the development of future clinical studies, with over 40 conducted to date. Dentists were invited via email (May 31 and June 16, 2022) and social media (Twitter, Facebook, Instagram), targeting general dentists and endodontists. Ethical approval was obtained from the University of Alabama at Birmingham Institutional Review Board, and implied consent was provided by survey completion.

In the United Kingdom, the same Quick Poll questions were embedded within a broader 32-question online survey distributed via JISC between November 2023 and March 2024. An additional question was included to explore definitive management preferences for irreversible pulpitis compared to the US Quick Poll. The online survey was distributed using an open sampling approach through multiple professional networks and organizations. These included Local Dental Committees (LDCs) across the UK nations, a range of Practice-Based Research Networks (PBRNs)—namely the Wales, Northern Ireland, East Midlands, South London, Northern Dental, and Scottish Dental PBRNs—as well as the Clinical Teachers group at King’s College London. Additional dissemination channels included professional associations such as the British Endodontic Society and the British Society of Periodontology, and social media platforms, including Facebook groups for UK dentists and the UK endodontic community on Instagram. For LDCs, distribution was confirmed with the respective chairpersons, and a follow-up reminder was sent to ensure circulation. For all other networks and channels, the online survey was distributed twice to maximize reach and engagement. Ethical approval was granted by the University of Dundee in May 2023. This parallel distribution allowed for international comparison of clinical views and practices regarding therapeutic pulpotomy in primary care settings. The reporting of this study adheres to the STROBE (Strengthening the Reporting of Observational Studies in Epidemiology) guidelines, and the completed STROBE checklist is provided as supplementary material [17].

### Clinical scenario

Both the USA and the UK studies used the same clinical scenario presented below as part of their study design.

‘*A 20-year-old female presents to your practice as an emergency appointment with shooting pain on eating and drinking from lower left quadrant. She is not able to specify a tooth. The pain lasts for an hour and is not relieved by analgesics*.

**Fig. 1:**
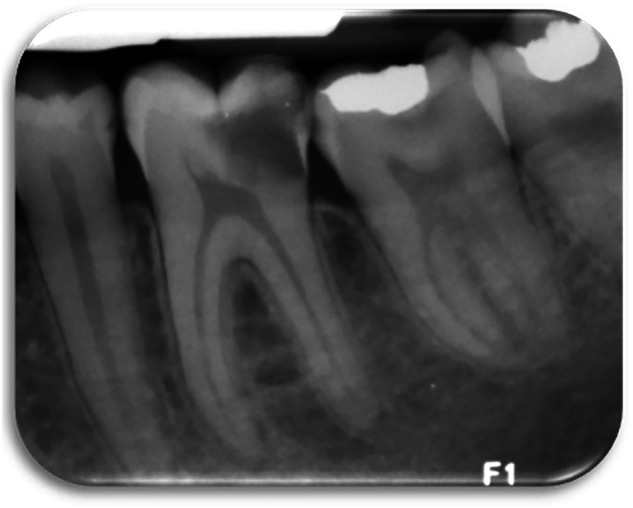
Periapical radiograph of lower left quadrant.

*Your oral examination reveals a carious lower left first molar, there are no sinuses or signs of swelling. The tooth is not tender to percussion. Cold testing revealed exaggerated response compared to the second molar, but the pain went away within 30-60 seconds. You take a radiograph which shows caries reaching the pulp and no apical radiolucency (*Fig. 1*). The pulpal diagnosis is an irreversible pulpitis and normal apical tissues in the lower left first molar*.’

The participants were then informed that this was a common clinical scenario illustrating an emergency presentation in a dental practice and that a new treatment option for such a case was emerging in the form of performing a full pulpotomy with tricalcium silicates on the tooth diagnosed with irreversible pulpitis and normal apical tissues. The participants were then briefly informed of the full pulpotomy technique namely *“removal of coronal pulp to the level of the canal orifices and achieving haemostasis before placing a tricalcium silicate cement such as MTA or Biodentine*^*TM*^ *in the pulp chamber followed by a definitive restoration.”*

Participants were asked to respond to the questions listed in Table 1. Additionally, they were asked whether they perform pulpotomy in their clinical practice. Data were collected via two secure online survey platforms: Constant Contact (USA) and JISC Online Surveys (UK). Descriptive statistics were used to analyse the data as the survey instruments and response options differed between the USA and UK cohorts, precluding direct inferential comparisons.

**Table 1:** Results of the USA and UK quick polls about irreversible pulpitis management.

	USA		UK	
	(*N* = 416)		(*N* = 334)	
	*N*	%	*N*	%
**Frequency of diagnosing irreversible pulpitis (per month)**				
0	0	0%	6	2%
05-Jan	218	52%	201	60%
10-Jun	103	25%	74	22%
>10	95	23%	53	16%
**Common clinical or emergency management for irreversible pulpitis**				
Prescribe or recommend analgesics	126	30%	85	25%
Prescribe antibiotics	83	20%	14	4%
Access the pulp and place a sedative temporary dressing	77	19%	288	86%
Perform a pulpotomy	83	20%	52	16%
Perform a root canal therapy	319	77%	87	26%
**Definitive management practice**s (**UK only**)				
Perform a pulpotomy			29	9%
Perform a root canal treatment			300	90%
Perform an extraction			167	50%
Refer to a specialist			56	17%
**Materials used for pulpotomy**				
Calcium hydroxide	101	44%	36	11%
Mineral trioxide agreggate (MTA)	55	24%	96	29%
Biodentine	37	16%	36	11%
Formocreosol	49	21%	0	0%
ZOE	42	18%	11	3%
Glass ionomers	44	19%	12	4%
I do not use pulpotomy as part of my clinical practice	0	0%	190	57%
Other	0	0%	10	3%
**Number of appointments required to complete molar root canal treatment**				
1 appointment	92	22%	32	10%
2 appointments	128	31%	201	60%
3 appointments	12	3%	83	25%
More than 3 appointments	0	0%	6	2%
I would refer the case to a specialist	132	32%	7	2%
I do not perform root canal treatment on any teeth	51	12%	5	2%
**Attitudes toward pulpotomy as a definitive treatment option**				
Both my patients and I would be in favour	182	44%	275	82%
Patients would be in favour but not myself	100	24%	13	4%
I would be in favour but not patients	12	3%	18	5%
Neither patients nor I would be in favour	118	28%	28	8%

### Ethical approval and consent to participate

The protocol for the Network -National Dental PBRN Administrative & Resource Center activities, which includes the Quick Polls were approved by the University of Alabama at Birmingham Institutional Review Board. Ethical approval for the UK arm of the study was obtained from the University of Dundee School of Dentistry Research Ethics Committee (UOD-SREC-SDEN-2023-009). Implied consent was obtained, where participants completed the Quick Poll and, by doing so, agreed to participate in the research. The study adhered to the Declaration of Helsinki.

## Results

### Response rate

USA Quick Poll: The survey was distributed via email and social media. The first email invitation achieved an open rate of approximately 40% with a 4% click-through rate, while a targeted follow-up email achieved 46% and 12%, respectively. In total, the survey page was accessed 1270 times, and 422 responses were submitted, including six via social media.

UK Quick Poll: The online survey was disseminated through LDCs, multiple PBRNs, professional associations, and social media platforms. As the survey was distributed using an open sampling approach across multiple channels, a response rate could not be calculated.

### Management of missing data

**Fig. 2:**
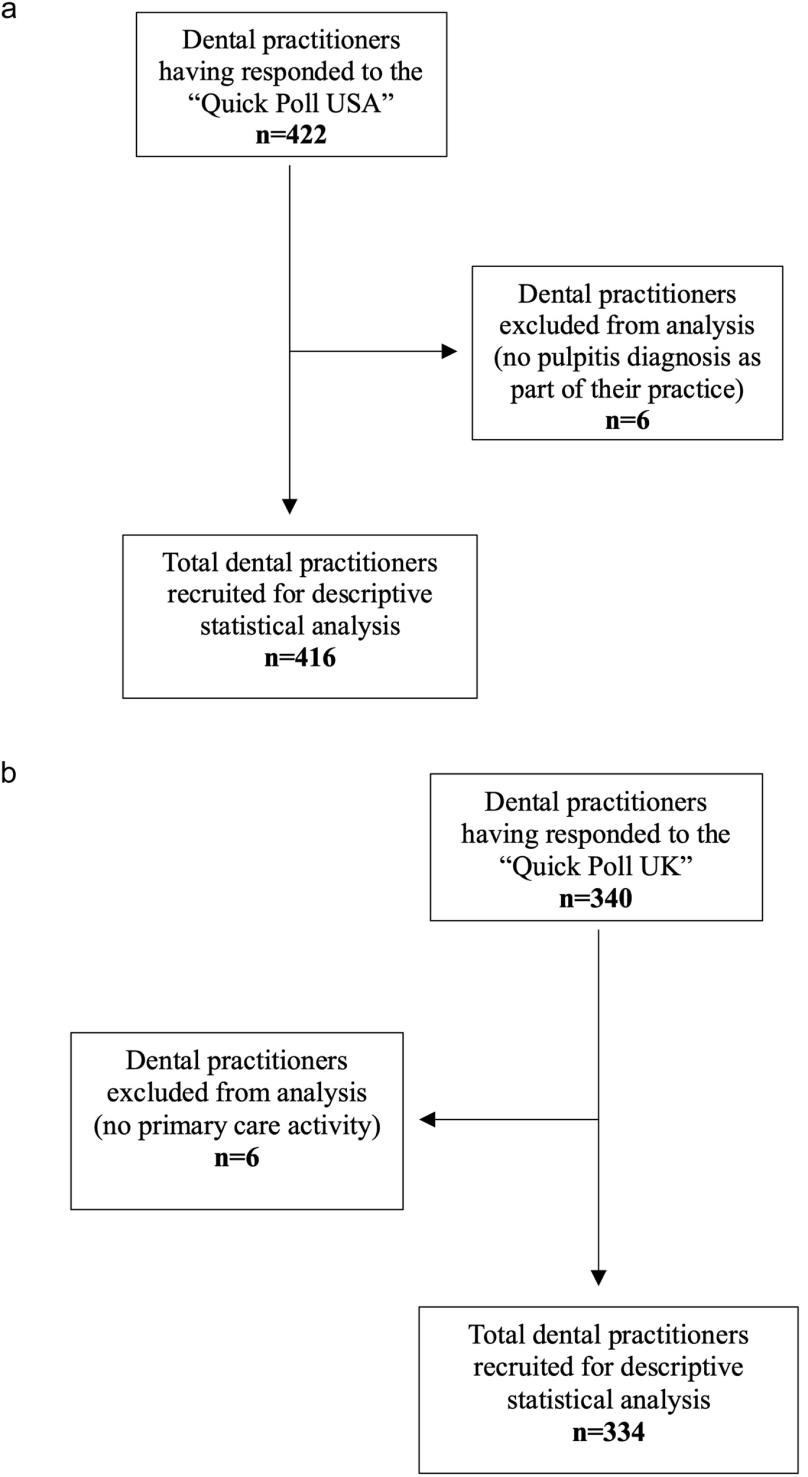
Flow of dental practitioner recruitment and exclusions for the USA and UK Quick Poll surveys. **a** STROBE flow diagram USA Quick Poll. **b** STROBE flow diagram UK Quick Poll.

USA Quick Poll: Of the 422 dental practitioners who completed the survey, six were excluded because they did not diagnose pulpitis in their practice, resulting in a final sample of 416 participants (10.8% of network members) (Fig. 2a).

UK Quick Poll: Of the 340 dental practitioners who completed the survey, six were excluded as they did not work in a primary care setting, leaving a final sample of 334 participants (Fig. 2b).

### Participants’ characteristics

The USA sample (*n* = 394) and UK sample (*n* = 334) differed in gender distribution (USA: 31% female; UK: 52% female) and age profile (USA: most aged over 50 years (63%); UK: most aged 31–50 years (56%)). Most respondents were general dental practitioners (USA: 89%; UK: 93%) with over 10 years in practice (USA: 85%; UK: 64%). In the UK, 68% worked in NHS primary care and 42% in private practice. In the USA, 84% worked in a private practice setting. Full demographic details are provided in Table 2.

**Table 2:** Participants’ reported characteristics

		USA (*N* = 416)		UK (*N* = 334)	
		*N* = 394^a^		*N* = 334	
		*N*	%	*N*	%
**Age**					
	21–30	7	2%	67	20%
	31–40	57	14%	100	30%
	41–50	79	20%	87	26%
	51–60	91	23%	64	19%
	>60	156	40%	12	4%
	Prefer not to say or missing	4	1%	4	1%
**Gender**					
	Female	122	31%	172	52%
	Male	271	69%	157	47%
	Prefer not to say or missing	1	0%	5	2%
**Years of practice since graduation**					
	0–5	15	4%	60	18%
	6–10	40	10%	60	18%
	11–20	82	21%	91	27%
	21–30	74	19%	69	21%
	>30	179	45%	53	16%
	Missing	4	1%		
**Current work situation**^b^					
	General practitioner	243	62%	303	82%
	General practitioner with enhanced training	108	27%	39	11%
	Specialist	38	10%	10	3%
	Currently in training	3	1%	14	4%
	Other or missing	2	1%	2	1%
**Practice setting**^c^					
	NHS			227	68%
	Private	329	84%	141	42%
	Community	28	7%	25	8%
	Other	37	9%	4	1%

^a^Information from 22 participants was missing.

^b^UK “dentists with special interest/enhanced skills” and US GPs with AEGD/GPR/specialty training are grouped as “GP with enhanced training.”

^c^NHS is UK-only, US community: safety-net and community health centers, and “Other”: academic, hospital, and federal/military settings.

### Diagnosis frequency

Most respondents reported diagnosing irreversible pulpitis in 1–5 patients per month (USA: 52%; UK: 60%), with fewer diagnosing 6–10 (USA: 25%; UK: 22%) or more than 10 cases (USA: 23%; UK: 16%) (Table 1).

### Management strategies

In the USA, root canal therapy was the most common management approach (77%), followed by analgesic prescription (30%), antibiotics (20%), pulpotomy (20%), and sedative dressing (19%). For emergency management, UK respondents primarily used sedative dressings (86%), with root canal therapy (26%) and pulpotomy (16%) less frequently reported. Antibiotic use was uncommon in the UK (4%) (Table 1).

### Definitive management (UK only)

For definitive care, UK practitioners most often performed root canal treatment (90%), followed by extraction (50%), referral (17%) and pulpotomy (9%) (Table 1).

### Pulpotomy use and materials

No USA respondents reported not using pulpotomy as part of their clinical practice. Meanwhile, over half of UK respondents (57%) reported not using pulpotomy in practice. Calcium hydroxide (44%) and MTA (24%) were the most common materials in the USA, whereas UK practitioners favoured MTA (29%) and Biodentine™ (11%). (Table 1).

### Root canal treatment appointments

USA respondents most often completed molar root canal therapy in two visits (31%) or one visit (22%), with 32% referring cases and 12% not performing RCT. UK respondents typically required two visits (60%) or three (25%), with referral and non-performance being rare (2% each) (Table 1).

### Attitudes toward use of pulpotomy

When dentists were asked whether they believed their patients and/or themselves would be in favour of pulpotomy for the management of irreversible pulpitis as an alternative to root canal treatment, almost half (47%) of USA respondents were in favour (44% were in favour and believed their patients would be in favour. 3% were in favour but believed their patient may not be), compared to 87% in the UK (82% were in favour and believed their patients would be in favour. 5% were in favour but believed their patient may not be). Similarly, 68% of USA respondents believed their patients would be favourable to pulpotomy, versus 86% in the UK (Table 1).

## Discussion

This study provides valuable insights into current clinical practices and attitudes toward the management of irreversible pulpitis across two large practitioner cohorts in the USA and UK. A key strength is the inclusion of a substantial sample size from both countries, allowing for cross-national comparisons and the exploration of demographic influences on treatment preferences. However, several limitations should be acknowledged. The survey relied on self-reported data, which may be subject to recall or social desirability bias. There is also an inherent bias in presenting the survey on a case with the diagnosis of “irreversible” pulpitis. Even though this is the currently accepted terminology, this term implies that the pulp is not likely to survive. In this regard, there are efforts underway to consider other terms for pulpal diagnosis [3, 18, 19]. The use of open sampling and multiple distribution channels precludes calculation of a response rate for the UK cohort and may introduce selection bias. Additionally, differences in question framing between the two surveys limit direct comparison of some responses, particularly regarding emergency management. Despite these limitations, the results highlight important trends and educational needs that can inform future research and guideline development.

The success of endodontic therapy relies on accurately assessing the degree of pulpal inflammation to determine the appropriate level of intervention. However, diagnosing pulpitis remains challenging due to the lack of objective criteria for correlating clinical symptoms with the histological status of the pulp [20, 21]. A significant part of respondents reported diagnosing irreversible pulpitis in six or more patients per month (USA: 48% and UK: 38%), underscoring the clinical relevance of this condition. The continued use of the term “irreversible pulpitis” implies it is a non-treatable pulp, despite growing evidence that many cases can be successfully managed with vital pulp therapy. Recent advances in materials and techniques have challenged this concept, yet terminology and consensus remain inconsistent [19]. The classification by Wolters et al., advocate for a gradient-based approach to pulpitis, ranging from “initial” to “severe,” to support minimally invasive strategies [3, 18, 19]. This framework recognizes that many pulps diagnosed as “irreversible” may retain healing potential when treated with conservative protocols, such as partial or full pulpotomy, particularly when inflammation is confined to the coronal pulp. Adoption of such classifications could influence treatment planning and align clinical practice with contemporary evidence favouring vital pulp therapy in selected cases.

Root canal therapy remains the predominant treatment choice for managing permanent molars with symptoms of irreversible pulpitis, reported by 77% of USA respondents and as the most frequent definitive treatment in the UK (90%). In contrast, pulpotomy was selected as a management strategy by only 20% of USA and 16% of UK respondents, despite the technical complexity and time demands of root canal therapy. This may be related to a perception among practitioners that there is insufficient evidence for the effectiveness of pulpotomy in cases of irreversible pulpitis. Some recent data show unfavourable long-term outcomes of vital pulp therapy [22]. Many practitioners reported completing root canal treatment over multiple appointments (most commonly two or more) or referring patients to a specialist, reflecting the perceived challenges of this procedure. Introducing a less invasive treatment option such as therapeutic pulpotomy for the management of irreversible pulpitis could benefit both patients and practitioners. This approach may reduce referral rates and increase access to endodontic care within the broader healthcare model.

Although calcium silicate-based materials have been available for decades [23], their routine use in vital pulp therapy remains limited. Our findings suggest that differences in age distribution between the USA and UK cohorts may partly contribute to variations in attitudes and adoption. The UK sample included younger practitioners, who are likely to have been exposed to contemporary evidence, minimally invasive approaches and use of calcium silicate-based materials during their undergraduate education, whereas the USA sample had a higher proportion of older practitioners, whose predoctoral training would have emphasized traditional techniques such as root canal therapy and use of calcium hydroxide.

Recent evidence from dental school curricula in the USA indicates that, although didactic teaching on vital pulp therapy is widespread, hands-on training opportunities remain scarce, with only one-third of schools offering simulated exercises [24]. This is very similar to the reported outcomes internationally (all schools teach about VPT but only 32.5% include practical elements of teaching) described by Nagendrababu et al., with 40 institutions across Europe, Asia, South America and Oceania [25]. These patterns, combined with previous research, indicate that barriers such as limited undergraduate exposure, lack of postgraduate training, material costs, and uncertainty about long-term outcomes continue to restrict the uptake of vital pulp therapies [26, 27]. Addressing these gaps through enhanced continuing professional development and integration of modern biomaterials into dental curricula is essential to support evidence-based practice moving forward and, in this context, adopting vital pulp therapies such as pulpotomy in permanent teeth in primary care. More broadly, this observation is consistent with implementation science literature suggesting that the translation of research evidence into routine clinical practice can take many years, with an often-cited average lag of approximately 17 years between evidence generation and widespread adoption, which may help contextualise the discrepancy observed between evidence and emerging guidelines [4, 9] and reported clinical practice in this study [28].

A notable difference between the theoretical acceptance of pulpotomy and its actual implementation in clinical practice was found. While only a minority of respondents reported pulpotomy as a common management strategy (USA: 20%; UK: 16%), a much larger proportion expressed willingness to consider it as a definitive treatment under appropriate conditions (USA: 47%; UK: 82%). Many practitioners seem to recognize the potential of vital pulp therapy but may face barriers such as limited training, uncertainty about long-term outcomes, and systemic factors like remuneration and material availability. The higher acceptance observed among UK respondents may reflect differences in educational exposure and healthcare delivery models, as well as a generational shift toward minimally invasive approaches as previously mentioned.

The results of the study highlight important trends and barriers in the clinical management of irreversible pulpitis, particularly regarding the adoption of vital pulp therapies. Moving forward, three key priorities should guide future research and implementation efforts. First, high-quality evidence from randomized controlled trials conducted in primary care settings is essential to test the effectiveness of pulpotomy as a definitive treatment, explore cost-effectiveness, and implementation strategies. The ongoing PIP Study in the UK exemplifies this approach, directly comparing pulpotomy and root canal therapy in general dental practice to assess clinical and economic outcomes [12]. Second, strengthening undergraduate and postgraduate education is critical to ensure that practitioners across generations are equipped with the knowledge and confidence to deliver evidence-based vital pulp therapy. Finally, providing practical tools—such as clinical decision aids [29], access to contemporary materials, and clear guidelines [4, 9]—will support primary care practitioners in integrating minimally invasive approaches into routine care. Together, these efforts can help bridge the gap between positive attitudes and clinical adoption, ultimately improving patient outcomes.

## Conclusion

This study provides a timely overview of current practices and attitudes toward the management of irreversible pulpitis in the USA and UK, highlighting both the persistence of traditional approaches and the emerging interest in vital pulp therapies. Differences in clinical adoption, educational exposure, and material preferences underscore the need for targeted efforts to support this evidence-based practice. By investing in high-quality research within primary care settings, enhancing educational pathways, and equipping practitioners with the tools needed to implement minimally invasive treatments, the dental profession can move toward more patient-centred and biologically driven care.

## Data Availability

The data that supports the USA findings of this study are available at https://www.nationaldentalpbrn.org/quick-polls/#1660317294621-b006b601-3efb.
