# Correction: Therapeutic pulpotomy for permanent teeth with irreversible pulpitis: comparative results from a practice-based quick poll in the USA and UK

**DOI:** 10.1038/s41405-026-00471-8

**Published:** 2026-08-07

**Authors:** Thibault NE Colloc, David NJ Ricketts, Janet E. Clarkson, Ashraf F. Fouad, Craig R. Ramsay, Joana Cunha-Cruz

**Affiliations:** 1 University of Dundee, Scotland, Dundee, UK; 2 University of Alabama, Birmingham, AL USA; 3 University of Aberdeen, Aberdeen, Scotland UK

**Keywords:** Dental epidemiology, Pulp conservation

Correction to: *BDJ Open* 10.1038/s41405-026-00404-5, published online 02 February 2026

In this article the author’s name Joana Cunha-Cruz was incorrectly written as Joana Cunha Cruz. Second, the affiliation, ‘University of Alabama at Birmingham, AL, USA.’ was incorrectly written ‘University of Alabama at, Birmingham, AL, USA’. The original article has been corrected.

